# Point-of-care ultrasound diagnosis of stump appendicitis in the emergency department

**DOI:** 10.1186/s13089-019-0128-5

**Published:** 2019-06-11

**Authors:** Apichaya Monsomboon, Bret P. Nelson, Phillip Andrus, James W. Tsung

**Affiliations:** 1 Division of Emergency Ultrasound, Department of Emergency Medicine, Icahn School of Medicine at Mount Sinai, New York, NY USA; 2 Present Address: Department of Emergency Medicine, Siriraj Hospital, Mahidol University, Bangkok, Thailand

## Abstract

**Background:**

Stump appendicitis (SA) is a rare entity in patients with a history of appendectomy and may result in missed or delayed diagnosis. We report a case of SA diagnosed by emergency department (ED) point-of-care ultrasound (PoCUS) in an elderly woman, thus expediting her care.

**Case presentation:**

An elderly female patient with a history of appendectomy 27 years ago was referred by her physician to the ED with right lower quadrant pain for 2 days. Using PoCUS the emergency physician identified SA. This was confirmed by computed tomography (CT) scan. The patient was then successfully managed non-operatively using antibiotics.

**Conclusions:**

Despite its rarity, it is feasible to diagnose SA using PoCUS, as patients presenting with right lower quadrant pain and history of appendectomy are at risk for delayed diagnosis, perforation, and poor outcome. PoCUS may reduce time to diagnosis, time to definitive operative or non-operative management, and minimize morbidity.

## Background

Stump appendicitis (SA) is a rare long-term complication of appendectomy. It is defined as the interval development of inflammation of the remaining appendix after an appendectomy [1]. The signs and symptoms are the same as acute appendicitis, thus making the diagnosis difficult. History of past appendectomy often leads to delayed or missed diagnosis that can cause morbidity. Perforation rate of SA reportedly ranges from 40 to 70% [1, 2].

SA was first described by Rose in 1945 [3], with a reported incidence of 1 in 50,000 cases [1]. However, the exact prevalence is not known [4] and likely under-reported as it may imply inadequate surgical technique [5]. Buttrick et al. [6] conducted a 5-year case review of 3252 appendectomies at our institution, which revealed only two cases of SA yielding a prevalence of approximately 0.06%. In a 60-year review by Subramanian and Liang, patients presented a mean of 108 ± 20 months after initial appendectomy [5]. It was unclear if there were specific high-risk features for the development of SA [5]. However, they noted in their review that SA occurred after both open and laparoscopic appendectomies. Additionally, they noted that the mean length of the residual stump was 3.3 cm (range 0.5–6.5 cm) with no patient reported to have SA with residual stump < 0.5 cm [5]. The most common presenting symptom was abdominal pain (93%) with 77% of patients having right lower quadrant pain [5]. Fifty-seven percent of patients presented with gastrointestinal symptoms, including nausea and vomiting [5]. The differential diagnoses of right lower quadrant abdominal pain in patients with history of appendectomy are listed in Table 1 [4].

**Table 1:** Differential diagnosis of right lower quadrant pain after appendectomy

Meckel’s diverticulitis	Psoas abscess
Cecal diverticulitis	Cholecystitis
Regional enteritis	PID
Leaking aneurysm	Torsion of ovarian cyst
Abdominal wall hematoma	Mittelschmerz
Ureteral stone	Endometriosis
Seminal vesiculitis	Mesenteric adenitis
Prostatitis	Right lower lobe pneumonia
Acute testicular disease	Appendiceal stump abscess

We report the first case of SA identified by point-of-care ultrasound (PoCUS) and was successfully managed non-operatively.

## Case report

A 69-year-old female was referred to the emergency department (ED) by her primary care physician with right lower quadrant abdominal pain for 2 days. She had nausea but no vomiting, fever, or diarrhea. She reported a history of appendectomy 27 years ago. Her past medical history included rheumatic heart disease with prolapsed mitral valve and hypothyroidism.

Physical examination revealed a temperature of 35.8 °C (96.4 °F), with otherwise stable vital signs. She had appendectomy scar and tenderness in the right lower quadrant without guarding or rebound tenderness. Her white blood cell count was 7.4 × 10^9^/L with neutrophils 62.5%.

**Fig. 1:**
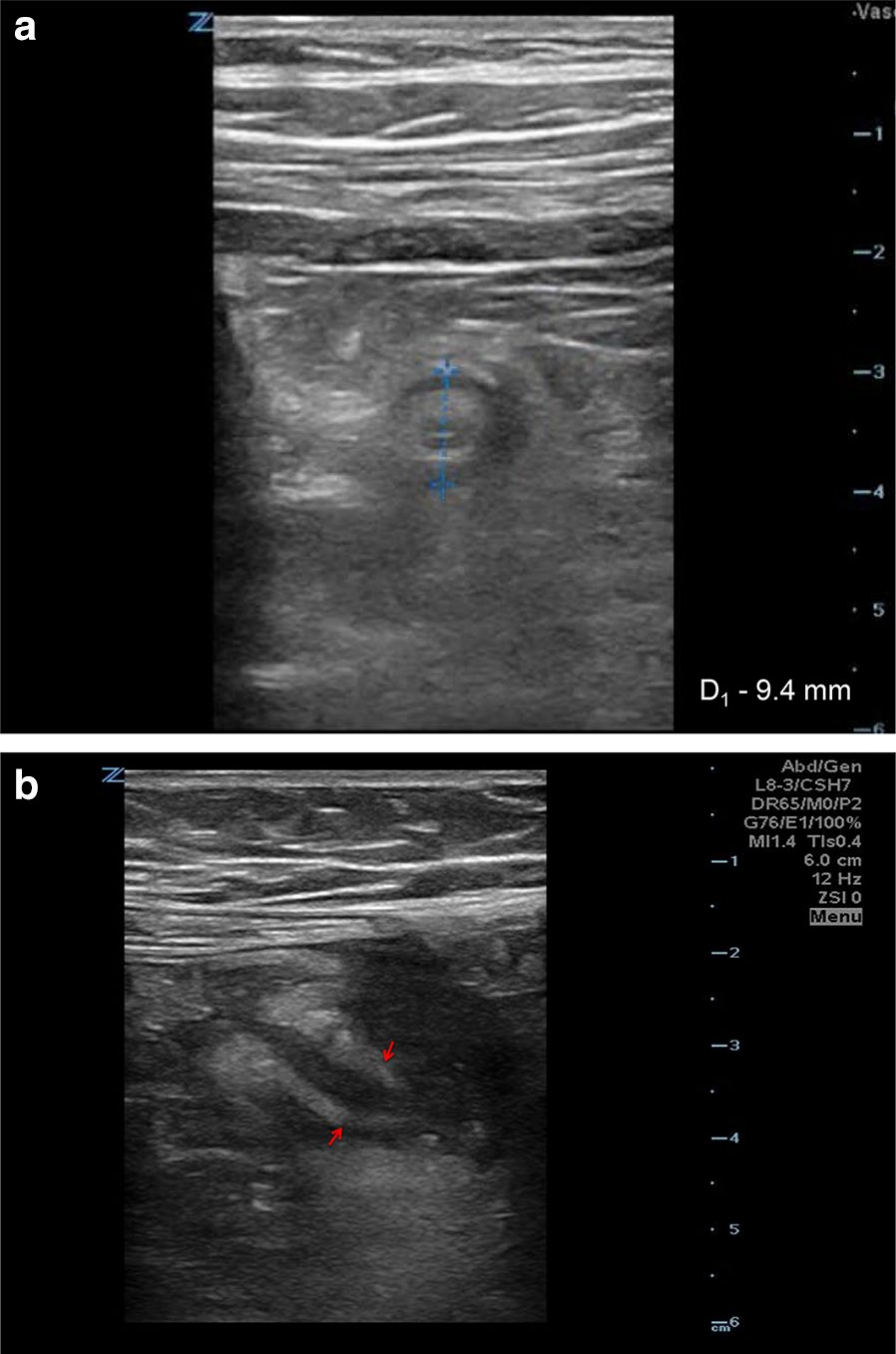
**a** Transverse (short axis) ultrasound scan shows SA measuring 9.4 mm in diameter. **b** Longitudinal ultrasound scan shows tubular blind-ended structure with surrounding with free fluid

**Fig. 2:**
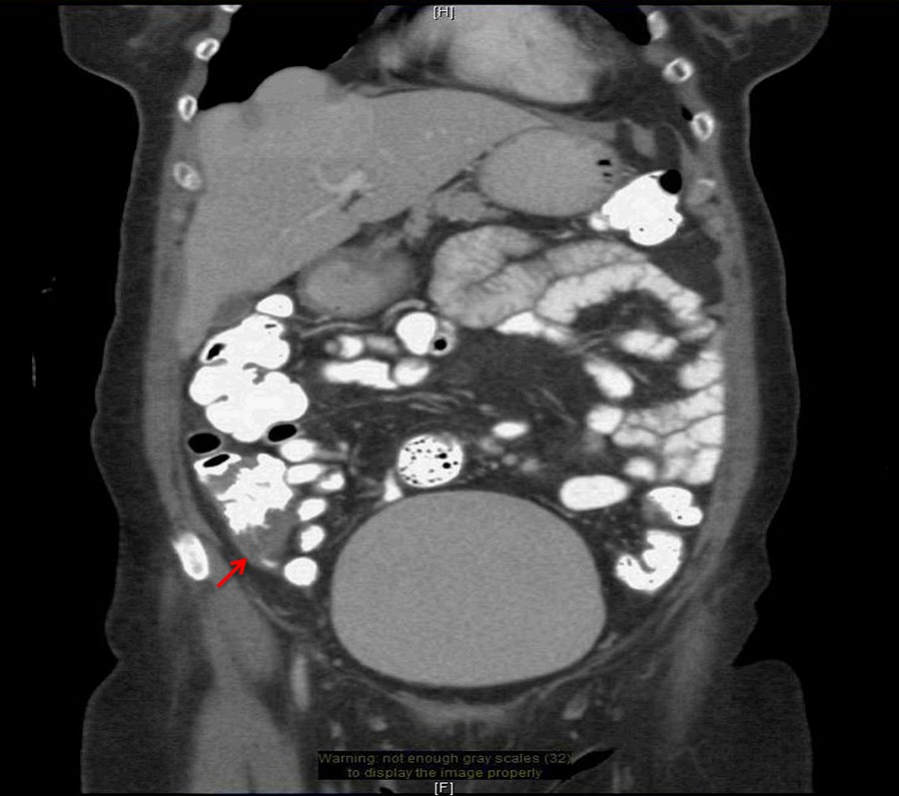
CT scan shows thickening and inflammatory change of a tubular structure extending from the base of the cecum, surrounding with inflammatory change

PoCUS demonstrated a non-compressible blind-ended tubular structure measuring 9.4 mm in diameter (Fig. 1a) connecting to cecum, surrounding with small amount of free fluid and fat stranding (Fig. 1b). Subsequent computed tomography (CT) scan of the abdomen and pelvis confirmed these findings (Fig. 2). Video link: https://youtu.be/BH5VrGYcfYc.

The patient was admitted and administered intravenous piperacillin–tazobactam. Given her history of rheumatic heart disease, non-operative management was chosen. One day after admission, her clinical examination significantly improved with no peritoneal signs. She was discharged with a 10-day course of oral levaquin and metronidazole. She had a follow-up CT scan 2 months later which was unremarkable.

## Procedure/technique

In patients who have localized right lower quadrant pain with high clinical suspicion for appendicitis, we recommend scanning the area of maximal tenderness and/or pain using a high-frequency (10–15 MHz) linear transducer. If the appendix is visualized, we try to identify the entire length of the appendix (abnormal cutoff diameter > 6 mm) from tip to base entering the cecum. We demonstrate the appendix in perpendicular orthogonal views (short and long axes). If the pain is non-localizing or more diffuse, we recommend starting at McBurney’s point, visualizing the right psoas muscle, identifying a loop of ileum, and then following it to the ileocecal valve and cecum which is often adjacent to the appendix. An enlarged appendix with abnormal cutoff diameter > 6 mm suggests SA. Additionally, compressibility of the SA relative to adjacent bowel is assessed by graded compression [7] or sonopalpation with the ultrasound transducer [8, 9].

## Discussion

To our knowledge, this is the first report of SA diagnosed with ED PoCUS. Before the advent of CT scan, definitive preoperative diagnosis of SA was not possible [10]. While CT scan may also diagnose other causes of abdominal pain (Table 1), it may prolong ED length-of-stay [8] and may not be available in low-resource settings. Since Puylaert et al. first described the graded compression technique for diagnosing appendicitis in 1986 [7], radiologist-performed ultrasound for SA, but not PoCUS, has been reported in the literature [5] as having the same as acute appendicitis. Published literature shows that PoCUS by emergency physicians has high specificity (97%) [11] and positive predictive value (91%) [11] to rule-in appendicitis [8, 11–13]. PoCUS can decrease ED length-of-stay compared to radiologist-performed ultrasound and CT scan, avoids radiation [8], and may be performed serially in non-operative management. One literature review of 51 reported cases of SA indicated that radiologist-performed ultrasound may well have a high accuracy in establishing the diagnosis of SA as it does for acute appendicitis, but no studies on sensitivity and specificity for SA have been published [14]. Nevertheless, the choice between ultrasound and CT in this clinical setting is largely dependent on institutional preference and available expertise [5, 14, 15].

A treatment of choice for SA is completion appendectomy either by open or laparoscopic intervention [6, 16]. There is one reported SA case that was successfully treated with non-operative treatment [2] as was our case. However, Hendahewa et al. reported a case of SA which was managed operatively initially, but developed recurrent SA again 3 years later, and subsequently underwent laparoscopic appendectomy [17]. Non-operative management for SA may be suitable for some patients, especially in those patients having multiple comorbidities or at risk for poor outcomes during surgery. Close follow-up in these patients is warranted, as concern for recurrent SA may approach recurrence rates in non-operatively managed appendicitis.

## Conclusion

Despite its rarity, it is feasible to diagnose SA using PoCUS, as patients presenting with right lower quadrant pain and history of appendectomy are at risk for delayed diagnosis, perforation, and poor outcome. PoCUS may reduce time to diagnosis, time to definitive operative or non-operative management, and minimize morbidity.

## Data Availability

Not applicable.

## Abbreviations

SA: stump appendicitis
ED: emergency department
CT: computed tomography
